# Using family history information to promote healthy lifestyles and prevent diseases; a discussion of the evidence

**DOI:** 10.1186/1471-2458-10-248

**Published:** 2010-05-13

**Authors:** Liesbeth Claassen, Lidewij Henneman, A Cecile JW Janssens, Miranda Wijdenes-Pijl, Nadeem Qureshi, Fiona M Walter, Paula W Yoon, Danielle RM Timmermans

**Affiliations:** 1 Department of Public and Occupational Health, EMGO Institute for Health and Care Research, VU University Medical Center, P.O. Box 7057, 1007 MB, Amsterdam, The Netherlands; 2 Department of Public Health and Department of Epidemiology, Erasmus MC, Dr. P. O. Box 2040, 3000 CA Rotterdam, The Netherlands; 3 Division of Primary Care, Graduate Medical School, University of Nottingham, Derby City General Hospital, Uttoxeter Road, Derby DE 22 3DT, UK; 4 General Practice and Primary Care Research Unit, Department of Public Health and Primary Care, Institute of Public Health, University of Cambridge, Robinson Way, Cambridge CB2 0SR, UK; 5 Division for Heart Disease and Stroke Prevention, Centers for Disease Prevention and Control, 4770 Buford Hwy, NE Atlanta, GA 30341, USA

## Abstract

**Background:**

A family history, reflecting genetic susceptibility as well as shared environmental and behavioral factors, is an important risk factor for common chronic multifactorial diseases such as cardiovascular diseases, type 2 diabetes and many cancers.

**Discussion:**

The purpose of the present paper is to discuss the evidence for the use of family history as a tool for primary prevention of common chronic diseases, in particular for tailored interventions aimed at promoting healthy lifestyles. The following questions are addressed: (1) What is the value of family history information as a determinant of personal disease risk?; (2)How can family history information be used to motivate at-risk individuals to adopt and maintain healthy lifestyles in order to prevent disease?; and (3) What additional studies are needed to assess the potential value of family history information as a tool to promote a healthy lifestyle?

**Summary:**

In addition to risk assessment, family history information can be used to personalize health messages, which are potentially more effective in promoting healthy lifestyles than standardized health messages. More research is needed on the evidence for the effectiveness of such a tool.

## Background

Clinical trial evidence shows that lifestyle modifications (e.g. weight loss, eating more healthily, increased physical activity and smoking cessation) can reduce the incidence of cardiovascular diseases [1], type 2 diabetes [2,3] and some types of cancers [4]. However, it has become increasingly clear that general health education programs aimed at the whole population have limited effect [5]. One way of increasing the effectiveness of these programs is to target interventions at individuals who are at increased risk of developing these diseases.

Most common chronic diseases are multifactorial in nature, resulting from interactions of genetic susceptibility, and behavioral and environmental influences. Susceptibility genes for common chronic diseases are newly discovered each day and advances in screening technology make it possible to screen large populations for multiple susceptibility genes [6]. Currently, several commercial companies, mostly in the US, already offer genetic profiling to the public (for an overview, see [7]). These companies claim that genetic profiling can provide accurate information about a person's susceptibility to a range of diseases. They suggest that genetic profiling can support decisions concerning preventive actions, including lifestyle choices [8]. However, for most genes included in the commercial profiles, the evidence for significant gene-disease associations is insufficient [9]. Hence, these tests currently have limited value in predicting disease risk and for developing personalized prevention messages.

Another important source of risk information to which most people have access is the health status of their close relatives (family history). Compared to genetic profiling, family history information has the advantage that it not only reflects the consequences of multiple genetic factors, but also captures the complex interactions between genetic, environmental, and behavioral factors, and may therefore be a better determinant of disease risk than genetic profiling. Epidemiological studies show that a family history is a strong and independent risk factor for cardiovascular diseases [10,11], type 2 diabetes [12], and many cancers [13-16]. In the clinical genetics setting, the value of systematically collecting and interpreting detailed family history information has long been recognized, i.e. for early diagnosis, decisions on genetic testing, and reproductive choices [17,18]. In primary care, the collection of family history information has been mainly used for diagnostic purposes in patients exhibiting disease symptoms, referrals for specialist evaluation (e.g. in the case of a suspected Mendelian disorder), and as a psychosocial tool to gain insight into family dynamics [19]. It has been suggested that the systematic collection and interpretation of family history may also be used as a tool for the prevention of common chronic diseases [20]. This information could not only be used to identify individuals at increased disease risk but also to raise risk awareness and motivate people to engage in risk-reducing behaviors. There is some evidence for the effectiveness of cancer screening on behavior following family history risk assessment [21,22]. However, only a few studies have examined the effectiveness of the use of family history information as a tool to promote a healthy lifestyle for primary disease prevention [23-26]. The purpose of the present paper is to discuss the evidence for the use of family history as a tool for primary prevention of common chronic diseases, such as cardiovascular disease, in particular for tailored interventions aimed at promoting healthy lifestyles. The following questions are addressed: (1) What is the value of family history information as a determinant of personal disease risk?; (2)How can family history information be used to motivate at-risk individuals to adopt and maintain healthy lifestyles in order to prevent disease?; and (3) What additional studies are needed to assess the potential value of family history information as a tool to promote a healthy lifestyle?

## Discussion

### What is the value of family history information as a determinant of personal disease risk?

In order to determine personal disease risk, clear methods to assess the risk associated with a given family history are required. To this end, epidemiological data need to be translated to individual risk. Several methods have been developed to facilitate interpretation of family history information. A comprehensive risk assessment tool that categorizes individuals into risk strata (e.g. low-, moderate- and high risk) based on family history, has been developed by Scheuner et al. [27]. To stratify individuals into risk groups, detailed information is collected about the number of affected family members, kinship and age of onset of a specific disease. Such tools have been developed for several multifactorial diseases. For example, as part of a public health initiative, the Centers for Disease Control and Prevention (CDC) developed an interactive multi-generational web-based tool to assess familial risk for six diseases (coronary heart disease, stroke, diabetes, and colorectal, breast, and ovarian cancer) [26]. Recently, three systematic reviews evaluating family history collection tools for clinical use have been published [28-30].

Besides risk stratification based on family history, this information can also be incorporated into a multifactorial risk assessment tool that includes other risk factors such as cholesterol levels and overweight. Examples include the QDScore, an algorithm to calculate diabetes risk [31], and the Reynolds Risk Score for cardiovascular disease risk assessment [32]. Family history information is generally assessed with a single yes or no question, asking if any one of the first degree family members has the disease. To each factor associated with the disease, appropriate weights, based on sound epidemiological evidence, are assigned. This multifactorial risk assessment approach has also been incorporated into several guidelines, for example European guidelines on diabetes, pre-diabetes, and cardiovascular disease [33].

In order to use family history information as a determinant of an individual's disease risk, the accuracy of self-reported family history should be ascertained. Studies show that for breast, colorectal and prostate cancers [16,34], as well as for cardiovascular diseases [35,36], relatives report with a reasonable degree of accuracy on the disease status of their close family members. The family history of other common chronic diseases, such as type 2 diabetes [37] and ovarian cancer [16,34], and less close family members (second degree relatives) are often reported with a lower degree of accuracy. The accuracy of self-reports is restricted by awareness and understanding of a condition in a family member. Cultural variation in how family is conceptualized can also affect the accuracy [38]. For example, in many cultures individuals place greater importance on, and have greater knowledge about, one side of the family. Raising public awareness of the importance of family history of cardiovascular diseases, type 2 diabetes and specific types of cancers (e.g. by mass-media campaigns) is likely toincrease the accuracy of self-reporting of family history information. Assuming that it is possible to collect fairly accurate information about a person's family history, more studies are needed to establish how this information should be interpreted to determine a person's disease risk, and by which methods for translating this information into individual risk assessment are most useful.

### How can family history information be used to motivate at-risk individuals to adopt and maintain healthy lifestyles in order to prevent disease?

Individuals who are at higher than average or population risk can be offered interventions to reduce or manage disease risk. In addition to medical recommendations, such as genetic testing, early screening (e.g. mammography) and medication, interventions may be aimed at promoting a healthy lifestyle. Often such interventions incorporate standard health messages (such as lose weight, eat more healthily, be more active, and stop smoking). However, the effectiveness of providing individuals who are at higher risk with these type of health messages is limited [5]. Individualized messages tailored to specific characteristics and knowledge of individuals with an increased risk can be more persuasive than standardized ("one-size fits all") messages [5,39,40].

The identification of health and illness beliefs that contribute to an individual's perception of disease risk may help to determine the key elements for tailoring individualized health messages. Most people with a family history have at least some relevant beliefs and knowledge about their disease risk. According to the Common Sense Model of the self-regulation of health and illness, these fragmentary beliefs are assembled into a mental model of personal disease risk (or illness representations), which people use in interpreting information [41,42]. Illness representations of people with a family history of a common disease can conflict with the epidemiological risk models of health professionals [43,44]. While lay understanding of personal disease risk may be based upon factors similar to epidemiological knowledge-such as the number of affected relatives, their age at diagnosis, and the level of kinship-other important factors have also been identified [44-50]. These include the experience of a relative's illness, feelings of closeness to the affected relative, and perceived differences between themselves and the affected relative (e.g. gender, age, personality, lifestyle and physical characteristics). Illness representations include beliefs about disease causation and controllability, in particular the influence of genetic and behavioral factors. Often people are not aware that their family history places them at risk. The role of behavioral factors may also be misunderstood. People not only tend to underestimate energy intake and overestimate physical activity [51] but also underestimate the potential consequences of maintaining their current lifestyle, and have little knowledge about preventive options [46]. Consequently, people may not be aware of a potential health problem and therefore may not see a need to change.

Prevention programs may be more likely to succeed if they incorporate an exploration of individual pre-existing illness representations alongside epidemiological risk factors [52]. Since health messages can be tailored to key elements of people's illness representations, one of the main challenges is to develop messages that fit within people's illness representations [53]. These messages should not only inform people about their disease risk based on what people already know and particularly addressing erroneous beliefs and gaps in knowledge, but should also motivate them to change their lifestyle.

To inform people about their disease risk, it is important that people receive objective and clear feedback on the risk associated with their family history and other risk factors. Often, risk information is communicated using verbal (e.g. high vs. low risk), numerical (frequencies and percentages), or visual (e.g. bar charts and icons) formats. These formats influence the effects on risk perception and subsequent behavior [54-59]. Besides probability information, people need to receive information about the nature of the risk (causes, consequences and severity) [55], because this information may make people more aware of the consequences of maintaining their current lifestyle, and broaden their illness representations to incorporate a multi-causal explanation of disease risk. Subsequently, individualized prevention messages can be offered that match these explanations, thereby increasing people's confidence in the effectiveness of specific prevention options [42]. For example, providing smokers with a family history of cardiovascular disease with an explanation of how smoking increases their risk may strengthen their confidence in the effectiveness of smoking cessation in reducing their risk for cardiovascular disease.

There is conflicting evidence about whether being a member of a family with affected relatives already has a positive effect on motivation to adopt a healthy lifestyle [60-62]. Few studies have assessed the effectiveness of using family history information as a tool to communicate disease risk and to motivate at-risk individuals to change lifestyles and maintain healthy ones. One study evaluating the use of a family history assessment tool reported increases in yearly medical examinations and blood pressure checks in both high- and average-risk families but did not report on lifestyle modifications [23]. According to a cross-sectional study, people who were informed of their familial risk of diabetes (by their doctors) reported greater awareness of risk and lifestyle changes to reduce risk, such as diet and exercise, than those who were not informed of their familial risk [24]. In a randomized controlled trial among high-risk individuals with a positive family history of type 2 diabetes, participants who had received additional information on familial risk reported more personal control over preventing diabetes and also reported more healthy eating habits than those who did not receive the additional information [25]. A large clinical trial assessing the clinical utility of a family history tool developed by the CDC is currently being evaluated [26]. In this multicenter trial, the effects of the provision of both familial risk assessment and prevention messages on risk perceptions, disease-related attitudes and beliefs, as well as change in health behaviors on members of primary care practices in the U.S. are examined.

### What additional studies are needed to assess the potential value of family history information as a tool to promote a healthy lifestyle?

A research agenda to evaluate the use of family history information in the prevention of common chronic diseases is currently being carried out in many governmental and academic institutions in the U.S. and elsewhere. This agenda addresses the key elements for assessing the added value of family history information, including the validity and interpretation of the information collected, and the practical barriers to implementation (see also [63,64] for an overview of specific research questions). However, this agenda does not specifically address how family history information can be used to motivate people to adopt and maintain a healthier lifestyle. To address the questions that were raised in this discussion, the research agenda should be refined and/or expanded.

In order to develop more effective personalized health messages that fit within people's mental model of disease risk, further research is needed into their pre-existing illness representations. In addition to qualitative studies, which can provide insight into the content of lay beliefs about personal disease risk, quantitative studies are needed to assess how these beliefs are assembled into a mental model and how they affect preventive behavior, for example how beliefs about family history and disease causation are linked to beliefs about the effectiveness of preventive options. More studies are also needed to assess whether and how these beliefs differ across individuals. Prior research has shown that beliefs vary across diseases and gender [65-67], but little is known about cultural and ethnic diversity and the effects of educational level [49].

In order to motivate people to adopt a healthier lifestyle, it may be possible to build on interventions that already have proven successful in changing lifestyle in high-risk individuals [1-4], such as in an extended follow-up study evaluating the effects of individualized counseling on lifestyle goals, such as reducing weight and increasing physical activity, in people at risk for diabetes [3]. Further research is needed to explore the effectiveness of differentiation of health messages based on family history information (high vs. low familial risk), other risk factor information, and to identify the components of the intervention that are most effective in achieving permanent lifestyle changes. It should also be noted that although providing information is usually a necessary prerequisite for behavior change, it is rarely sufficient to promote change. When designing interventions other determinants, such as the social and physical environment, need to be considered.

In addition, more knowledge is needed about the best mode of delivery of family history risk information. Physicians have reported time restrictions, lack of reimbursement, and the complexity of familial risk interpretation as barriers to the routine and systematic collection and use of family history for disease prevention [68]. Decision support systems and computer-aided tools that can be self-administered or administered by a nurse practitioner or physician assistant might reduce some of these barriers. In addition, internet-based interventions have the potential to reach more people at lower costs. However, they lack the social support that individuals receive from interpersonal counseling, while disparities in computer skills, internet access and public concerns about internet security may affect response.

Finally, there is the possibility that knowledge of family history will have adverse psychological effects. Some experts consider that informing people about an increased risk based on their family history could induce a sense of fatalism-the belief that little can be done to change the risk-which can decrease motivation to change behavior [53,69,70]. The family history risk information may also evoke anxiety, which can induce maladaptive responses, such as avoidance or denial of the presented information [71]. False reassurance, either because a person has no family history or does not identify with the affected relative, is another potential adverse effect that needs careful examination [72]. So far, in studies where familial risk was discussed with people who had a family history of diabetes [25,73,74] and colorectal cancer [75,76] no (long term) adverse psychological effects were noted. This is in line with the larger literature on the emotional impact of a wide range of health risk assessments (see review by Shaw et al. [77]).

## Summary

Detailed family history information can be used-along with personal risk factors such as weight and smoking status-as a simple, easily applied and cost-effective tool to determine a person's disease risk. In addition to risk assessment, family history information can also be used to personalize health messages, which may be more effective in motivating people to adopt and maintain a health lifestyle than standardized health messages. A personalized health message should be phrased in such a way that it fits within the target's pre-existing beliefs about current health status, possible causes and risk factors, age of onset and course of the disease, magnitude of and potential consequences of the risk, and ways to reduce the risk. In this way, personalized risk information can correct erroneous beliefs, fill knowledge gaps, and reinforce people's confidence in their ability to change behavior. The evidence for the effectiveness of using family history information as a personalized tool for disease prevention, in particular for raising motivation to adopt and maintain a healthy lifestyle, is very limited. More research is needed before a definite answer can be given to the question of whether and how family history information should be used and promoted as a tool to motivate people to change their behavior.

## Competing interests

The authors declare that they have no competing interests.

## Authors' contributions

All authors participated in the conception of this overview and have been involved in the drafting of the manuscript. All authors read and approved the final manuscript.

## Pre-publication history

The pre-publication history for this paper can be accessed here:

http://www.biomedcentral.com/1471-2458/10/248/prepub
